# A rare case of a intraosseous arteriovenous malformation of the temporomandibular joint and mandible – Case report and literature review

**DOI:** 10.1016/j.ijscr.2020.10.011

**Published:** 2020-10-07

**Authors:** Kristian K. Blackhall, Eugenie Ling, Jayanth Kunjur

**Affiliations:** Dept. Oral and Maxillofacial Surgery, Salisbury District Hospital, Salisbury, Wiltshire, SP2 8BJ, UK

**Keywords:** AVM, arteriovenous malformation, Maxillofacial, Arteriovenous malformation, Temporomandibular joint replacement, Case report

## Abstract

**Introduction:**

This case report looks at a 47 year-old patient, presenting with a rapid onset mass of the left face. This report examines the case, the investigations undertaken, its management and undertakes a literature review of this uncommon condition and rare presentation.

**Presentation of case:**

This patient had experienced functional deterioration, restriction and an evident swelling of the left face. Having identified the mass to arise from the mandibular condyle, an incisional biopsy was undertaken. The histology was suggestive of an arteriovenous malformation. Surgery was undertaken to excise the entire left mandibular condyle along with the lesion and then replace the temporomandibular joint with a custom made prosthesis.

**Discussion:**

Arteriovenous malformations, or Haemiangiomas, can arise in any part of the body and can often grow over time. Although benign, lesions can be locally invasive and distort anatomy, resulting in functional issues. Their risk for hemorrhage and potentially significant blood losses is also a risk factor in their presence. Rarely arteriovenous malformations can occur in hard tissue, as in this case distorting and obliterating the temporomandibular joint. One’s only recourse is to remove and eliminate the lesion, however in cases such as this significant morbidity could ensue due to functional deficit. Hence consideration must be given to reconstruction.

**Conclusion:**

Having excised the mass and reconstructed the temporomandibular joint with a titanium prosthesis, the patient experienced a good recovery with a return to normality.

## Highlights

• Arteriovenous malformations (AVMs) are benign but locally invasive lesions.
• The mandible is a rare location of AVM, comprising less than 0.5–1% of all lesions.
• An AVM presentation within the temporomandibular joint is highly unusual.
• Reconstruction with titanium prostheses allows for excellent postoperative function.

## 1 Introduction

Arteriovenous malformations (AVMs) are benign lesions that can arise in any part of the body [1]. They are formed through abnormal connections of arteries and veins, often developing to resemble a mass initially similar to a tumour. Rarely, AVMs can arise within hard tissue structures such as bone/jaws and give rise to a highly unusual spectrum of symptoms and presentations [2].

AVMs lack the dampening effect that capillaries would usually have on blood flow, as a result they can become progressively larger over time, with the surrounding tissues becoming deprived of blood flow and suffering ischaemic effects. The resulting tangle of blood vessels is often termed a “nidus” and can be extremely fragile and prone to bleeding due to the abnormal direct connections between high-pressure arteries and low-pressure veins [3].

AVMs can be asymptomatic and are generally detected due to causing resultant effects on surrounding structures. Within the jaws AVMs are very rare, with a prevalence of less than 1/100,000 person years [1]. Often diagnosis can be challenging and radiological features variable possibly resulting in confusion with cystic lesions or tumours [4].

## 2 Presentation of case

This case pertains to a 47 year-old causacian female who presented to the Oral and Maxillofacial Surgery Department at Salisbury NHS Hospital. The patient was otherwise medically fit with no significant medical, drug or familial history.

The presenting complaint involved non-painful swelling of the left cheek/preauricular region that arose over a couple of weeks. There was no history of injury to the area, nor any discharge or associated rubor/calor/dolor. The lesion was firm and appeared to distend the overlying tissues with the appearance of arising from deeper structures. Intra-oral examination did not reveal any dental pathology and salivary expression was normal.

In a differential diagnosis, a lesion of the parotid salivary gland was amongst the more likely potential explanations. Physical examination alone was insufficient to determine the lesion’s origins, hence CT scanning was undertaken.

**Fig. 1:**
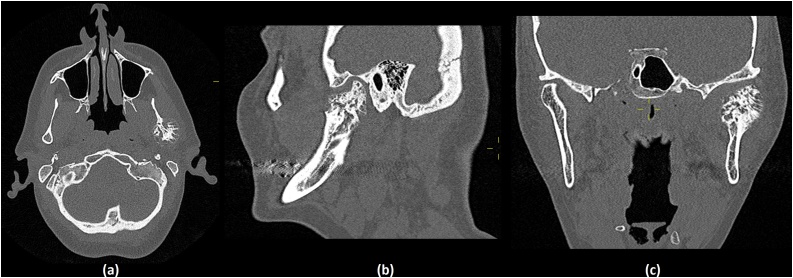
CT Imaging demonstrating the lesion of the left temporomandibular joint and mandible. (a) - Axial plane, (b) - Sagittal plane, (c) - Coronal plane.

Imaging demonstrated a highly unusual lesion associated with the left temporomandibular joint. The lesion was well circumscribed, confined to the mandibular condyle, partially lytic in appearance and did not appear to be causing any destructive effects on the surrounding tissues. The overall size was large, measuring at 32 × 23 × 29 mm. Generally the appearances were benign, however a firm diagnosis could not be achieved. As such the decision was taken to perform an incisional biopsy (Fig. 1).

**Fig. 2:**
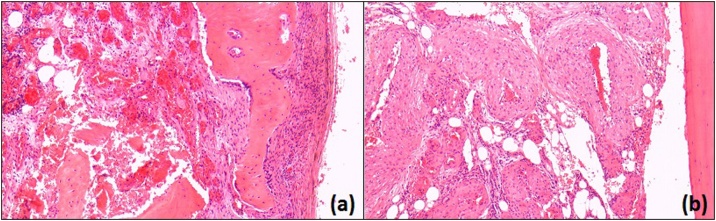
Histology slides obtained. (a) - Demonstrating capillary structures [2.5× power], (b) - Demonstrating larger vascular structures [2.5× power].

Samples of abnormal bone were obtained along with surrounding soft tissues. Macroscopic analysis revealed an irregular bony surface with distortion and expansive signs. The sample was decalcified for microscopic analysis. This showed areas of proliferative blood vessels between bony trabeculae, certain areas also showed leashes of small to medium-sized blood vessels which lacked elastin in their walls and were dilated. Much of the bone was replaced with these vascular structures. The remaining, expanded bony surface was covered with fibrous tissue. Two histopathology consultants agreed that these findings were consistent with an AVM (Fig. 2).

Following the International Society for Study of Vascular Anomalies (ISSVA) classification system, this AVM would be categorised as an Intraosseous Vascular Malformation (VMOS), under Simple Vascular Malformations Type III Category [5]. There were no feeder vessels and ultimately embolisation was not undertaken in the management of this lesion (see below).

Due to the expanding nature of the lesion and increasingly deleterious effects on the patient, the decision was taken to operate. The surgical plan being to resect the left mandibular condyle head, neck and a portion of the ramus, essentially obliterating the temporomandibular joint. Although curative intent would be achieved through this approach, the resulting effects on the patient’s quality of life and function would be significant. As such, consideration was given to reconstructive options available for such a resection. With the glenoid fossa and skull base unaffected, a custom joint replacement was thought to be the best option, restoring both normal anatomy and function.

**Fig. 3:**
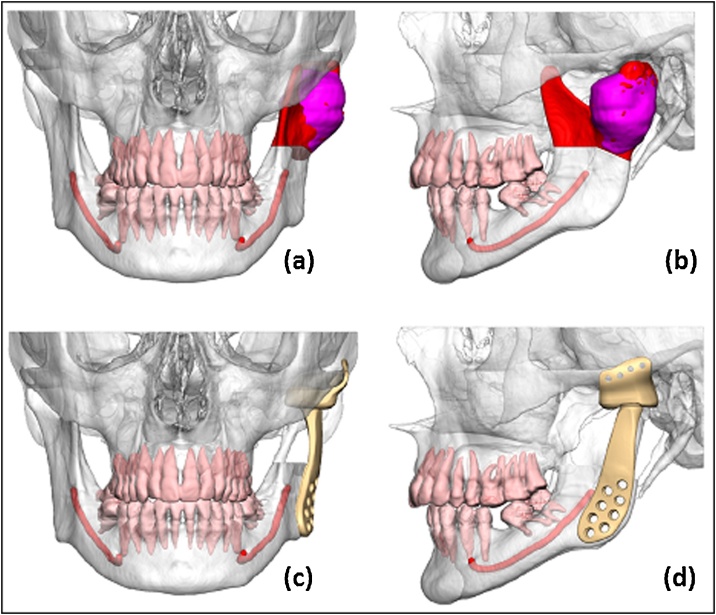
3D planning render for the left temporomandibular joint. (a)+(b) - Preoperative view demonstrating AVM extent [purple] and planned resection [red], (c)+(d) - Postoperative titanium prosthesis position.

Given the anatomical variations often seen with temporomandibular joints and their need to work in tandem with the opposite side, a custom prosthetic option was selected. This would allow for precise planning and replication of the anatomy, in what was likely to be a challenging and non-standard resection. The CT scans obtained allowed for digital planning, design and manufacture of the titanium prosthesis (Fig. 3).

Surgery was performed by a Maxillofacial team, including a consultant surgeon and specialty doctor surgeons, all team members were certified and held tri-collegiate fellowship/membership with The Royal College of Surgeons. An approach was selected to allow sufficient access for resection as well as placement of the prosthesis. A preauricular incision with temporal extension was done for initial access. This allowed for direct dissection down to the field. A second incision was made using a retromandibular approach to aid access.

Using a surgical guide, bony cuts were made so as to resect the affected field, leaving a portion of the ramus. As the AVM was completely intraosseous there was no uncontrollable haemorrhage. The prosthesis was then placed on the remaining bony surface of the mandibular ramus and fixated with bicortical screws. Throughout the procedure the patient was placed into rigid intermaxillary fixation to keep the jaws locked together, maintaining the occlusion of the teeth and the position of the temporomandibular joint of the opposite side. Layered closure was done with a drain placed to prevent accumulations. Following an uneventful recovery the patient was discharged the following day.

Recovery in the postoperative phase was routine with no complications or facial nerve weakness. Allowing for sufficient time, healing and oedema to subside at home, the patient returned to relative normality with their function and facial profile over 3 weeks, eating a normal diet with no discomfort. Postoperative imaging taken at 3 months indicated a good position of the prosthesis with integration to the mandible. The final histology supported the diagnosis of a benign AVM.

## 3 Discussion

Although AVMs of the jaw are rare, one should appreciate their part of a differential diagnosis when considering options for unexplained symptoms and pathology. Studies have shown that although AVMs have a risk of haemorrhage [6], intraosseous AVMs have an increased tendency of life-threatening haemorrhage either spontaneously or after surgery [4]. The aetiology of AVMs and particularly intraosseous presentations remain unclear, it is theorised that lesions can arise as a result of embryological abnormalities of differentiation at early stages of development [7] as well as having a genetic/familial link [6].

Given the slow-growing nature of AVMs they are often quiescent for a significant period and do not tend to show increased incidence for a particular age group [8]. With the complexity of the maxillofacial region, morphology of jaws and the presence of teeth, AVMs of the jaws are more difficult to identify and manage than other intraosseous AVMs [9]. Although rare, in the reported cases of maxillofacial intraosseous AVMs, there are several correlating features. A firm, painless, bony expansion is generally seen which can cause gross facial asymmetry and distortion of local anatomy, however pain and paraesthesia are not directly linked to AVMs but can occur due to expansion and insult of adjacent structures [10]. If occurring in proximity to teeth, AVMs can cause mobility and displacement, in keeping with their slow-growing nature. However, there have been reports of root resorption occurring [11], which can confuse diagnostics as this is often associated with more destructive disease processes such as ameloblastomas or osteosarcomas.

AVMs of the jaws are very rare, accounting for (0.5–1%) of all lesions [12]. Literature searches revealed only a select few reported cases, but it seems no cases of AVMs manifesting from within the temporomandibular joint have been reported.

Radiographic features of AVMs can be similar to other lesions so definitive diagnosis based solely on imaging is difficult [12]. Key radiographic features include parallel radio-opaque striations, altered radiodensity with osteolytic areas and a generally altered trabecular pattern [13]. The osteolytic areas are particularly key and a strong feature of AVMs, often described as a ‘honeycomb’ appearance [14]. The bony surface configuration can appear disrupted with scalloped areas and both unilocular or multilocular appearances as well as ‘sunburst’ effects [15]. Hence potentially adding further confusion, as these features can be associated with lesions such as osteosarcomas, ameloblastomas, myxomas or fibrous dysplasia [13,15].

Definitive diagnostics can only reliably be achieved through biopsy and histopathological examination. Features are fairly consistent in AVM histology, proliferative epithelial cells and fibrous tissue are often seen surrounding the lesion and the remaining bony trabeculae are interspersed with vascular structures and endothelial cells [16].

There are various treatment options available for AVMs, the key aims being; elimination, haemorrhage control and preventing recurrence. Some AVM lesions may be inaccessible and in these cases radiotherapy is the only treatment modality available. Cases have been reported of radiotherapy successfully controlling AVM lesions [17], however any bony deformity or effects on adjacent structures can only be controlled and corrected through surgery [17].

Direct surgery with or without embolisation of major afferent feeding vessels is the accepted treatment of choice [11]. Radical excision of the lesion allows for a curative intent and generally immediate reconstruction is undertaken with a tissue graft or, as in this case, a joint prosthesis. Embolisation alone is generally not recommended, but may be an appropriate option for patients who are not ideal candidates for more extensive surgery [18].

In this case, wherein the entire joint was excised, the use of a custom prosthesis was selected. Given the essential functions of mastication, speech, airway support and deglutition are directly supported by the temporomandibular joint, restoration of function was of principal concern. Indeed it has been shown that improvement of temporomandibular joint function with the use of a prosthesis results in an improved quality of life of up to 85% over the long term [19], and this extensive AVM presentation would be categorised as an ‘end-stage’ temporomandibular joint disorder.

## 4 Conclusion

Due to the complex presentation and multifactorial nature of AVMs a step-wise diagnostic approach should be undertaken. CT scanning can guide and aid in surgical planning, but would be inappropriate to solely rely upon for definitive diagnosis. Given the spectrum of imaging presentations seen and the crossover with other lesions, microscopic histological examination is the gold standard for diagnosis. Of the various treatment modalities available the primary goals remain; elimination, haemorrhage control and preventing recurrence. With reconstruction bearing importance depending on the functional deficits likely to be encountered through surgery.

This case has been reported in line with the SCARE 2018 criteria [20].

## Declaration of Competing Interest

The authors report no declarations of interest.

## Funding

We have no funding sources to report.

## Ethical approval

Institutional approval was obtained for the study. Ethical approval is not applicable to this submission type.

## Consent

Written informed consent was obtained from the patient for publication of this case report and accompanying images. A copy of the written consent is available for review by the Editor-in-Chief of this journal on request.

## Author contribution

**Blackhall, Kristian K:** Lead author & corresponding author. Design of report and literature review. Literature search. Data analysis. Writing of the paper.

**Ling, Eugenie:** Assisting in writing of the paper. Literature search and analysis.

**Kunjur, Jayanth:** Supervising consultant surgeon. Clinical lead for this surgical case. Writing of the paper.

## Registration of research studies

1. Name of the registry: N/A
2. Unique identifying number or registration ID: N/A
3. Hyperlink to your specific registration (must be publicly accessible and will be checked): N/A

## Guarantor

Blackhall, Kristian K.

## Provenance and peer review

Not commissioned, externally peer-reviewed.
